# Molecular insights into supercritical water gasification process of polyoxymethylene plastics

**DOI:** 10.1038/s41598-025-93887-5

**Published:** 2025-03-18

**Authors:** Tuong Ha Do, Dao Trinh, Thi Be Ta Truong, Thuat T. Trinh

**Affiliations:** 1 Group of Applied Research in Advanced Materials for Sustainable Development, Faculty of Applied Sciences, Ton Duc Thang University, Ho Chi Minh City, Vietnam; 2 Laboratoire des Sciences de l’Ingénieur pour l’Environnement, LaSIE UMR 7356 CNRS, La Rochelle Université, Avenue Michel Crépeau, 17000 La Rochelle, France; 3 Faculty of Applied Sciences, Ton Duc Thang University, Ho Chi Minh City, Vietnam; 4 Porelab, Department of Chemistry, Norwegian University of Science and Technology, NTNU, 7491 Trondheim, Norway

**Keywords:** Pollution remediation, Chemical engineering, Computational chemistry, Molecular dynamics, Reaction mechanisms

## Abstract

Effective plastic management is crucial in addressing the growing environmental challenges posed by plastic pollution. Among various plastics, polyoxymethylene (POM) stands out as a widely used engineering thermoplastic with significant applications in industries . Innovative recycling solutions are essential to mitigate its environmental impact. This study investigates the supercritical water gasification (SCWG) of POM plastics at a molecular level using reactive molecular dynamics simulations. The research aims to provide insights into the factors influencing the SCWG process. Key findings reveal that temperature significantly affects reaction mechanisms, while the primary syngas products include hydrogen , carbon monoxide, and carbon dioxide. A notable trend observed is the increase in activation energy as water content increases, highlighting the importance of optimizing hydration levels for efficient conversion. The calculated activation energies range from 106 to 135 kJ/mol, aligning well with experimental findings (160 kJ/mol). The study validates the computational approach by demonstrating excellent agreement between simulation results and experimental findings on the molar fraction of gas and activation energy, underscoring its reliability as a predictive tool for process design and optimization. Furthermore, the research contributes to sustainable waste management by offering strategies to enhance SCWG efficiency.

## Introduction

The global plastic pollution crisis is one of the most pressing environmental challenges today, driven by plastics’ widespread use in versatile applications^1^. Annually, 300 million tonnes are produced, much of which is short-lived packaging, overwhelming waste management systems and leading to ecosystem contamination^1^. Current strategies like recycling, landfilling, and incineration face significant limitations. Recycling struggles with contamination and high costs, while infrastructure gaps limit its effectiveness worldwide. Despite global efforts, plastic waste is projected to grow faster than mitigation measures^2^.

Plastics have revolutionized modern society due to their versatility, durability, and low production costs. However, the accumulation of plastic waste poses significant environmental challenges. Plastics persist in natural environments, impacting ecosystems and human health^3^. Weathering processes fragment larger debris into microplastics, which act as vectors for persistent organic pollutants (POPs), raising concerns about bioaccumulation and ecological damage. Microbial communities on marine plastic debris may contribute to degradation through hydrolysis and enzymatic processes, though further research is needed^4^.

Advancements in detecting microplastics require standardized approaches for effective monitoring^5^. Chemical additives in plastics release pollutants, posing risks to marine life and potentially entering the food chain^6^. Biodegradable alternatives like polylactic acid offer potential but face challenges in mechanical properties and degradation conditions^7,8^. Safer chemical additives are essential to minimize environmental contamination during use, disposal, and recycling^9^.

Sustainable solutions include biodegradable polymers and technologies like pyrolysis for resource recovery^10,11^. Improved waste management systems require integrating recycling, composting, and anaerobic digestion to enhance resource efficiency^12^. Additionally, standardized definitions and public awareness are crucial for effective policy-making, alongside addressing issues like phthalate additives^13,14^.

Polyoxymethylene (POM), also known as polyacetal, is a thermoplastic polymer renowned for its high strength-to-weight ratio, excellent dimensional stability, and wear resistance. Its structure consists of repeating ($-\hbox {CH}_{2}-\hbox {O}-$) units synthesized through formaldehyde polymerization^15^. Discovered in the 1930s, POM has found widespread use across industries^16–19^ due to its versatility. Hale et al. demonstrated POM’s utility as passive samplers for measuring bioavailable polycyclic aromatic hydrocarbons (PAHs) in biochars, aiding contaminant assessments^20^. In energy storage, Zhang et al. found that POM enhances the stability of solid-electrolyte interphases (SEI) in lithium batteries, improving performance and cycling efficiency^21^. Additionally, Jonker and Koelmans (2001) introduced a POM-based solid-phase extraction method for analyzing hydrophobic organic chemicals, enhancing environmental monitoring accuracy^22^. The global production of POM amounts to approximately 1.7 million tons annually^23^. If not managed properly, POM can contribute significantly to the growing plastic waste problem, exacerbating environmental challenges associated with pollution^24^. Developing effective recycling strategies for POM plastics is of paramount importance to mitigate their environmental impact.

Supercritical water gasification (SCWG) is an advanced thermochemical process that converts biomass into hydrogen and other valuable gases under high-temperature and high-pressure conditions. This technology operates in the supercritical region of water, where it exhibits unique properties as a solvent and reactant, enabling efficient conversion of organic matter into gaseous products. Recent studies have highlighted SCWG’s potential as a cost-effective method for hydrogen production from renewable biomass resources^25^. Unlike traditional gasification methods that require drying of feedstocks, SCWG can directly utilize high-moisture biomass, reducing preprocessing costs and enhancing operational efficiency.

Research by Zhang et al. underscores SCWG’s versatility in handling various biomass feedstocks, including agricultural residues and food wastes, to produce hydrogen-rich gas^26^. Their findings demonstrate that SCWG not only achieves high hydrogen yields but also minimizes tar and char formation, which are common challenges in conventional gasification processes. This makes SCWG a promising candidate for sustainable biohydrogen production. Technical advancements in reactor design have addressed several operational challenges associated with SCWG. Early studies by Antal Jr. et al. reported issues such as reactor plugging due to ash and char accumulation, which limited continuous operation^27^. However, subsequent research has focused on optimizing reactor geometries, catalysts, and operating conditions to enhance durability and efficiency. For instance, the integration of packed carbon beds within reactors has improved gasification efficiency by catalyzing the breakdown of organic vapors^28^. Recent research by Lu et al. has investigated the SCWG of POM plastics, examining how factors like temperature, residence time, feedstock concentration, and pressure influence gasification efficiency^29^. Their findings reveal that higher temperatures significantly enhance gasification performance, with a total carbon conversion rate reaching 99% at $700^{\circ }\hbox {C}$. This study underscores the potential of SCWG in effectively managing POM waste.

The use of molecular dynamics (MD) simulations to study reaction mechanisms and kinetics has become increasingly important in research^30–35^. Reactive force field (ReaxFF) molecular dynamics simulations are powerful computational tools for studying polymer behavior at the molecular level. They enable the simulation of chemical reactions and bond-breaking processes, making them ideal for investigating complex phenomena such as mechanical reinforcement and oxidative degradation in polymers^36^. For instance, Ha et al. employed the ReaxFF force field to investigate the gasification process of polyethylene, providing detailed insights into the reaction pathways and energy barriers involved in polymer degradation^37^. Another work by Islam et al. studied single-walled carbon nanotube reinforced POM composites, varying CNT diameters and volume fractions to observe improvements in mechanical properties^31^. Their stress-strain plots revealed enhanced strength and toughness, highlighting nanotechnology’s potential in polymer reinforcement. Chen et al. compared the oxidation resistance of polyethylene and POM in hydrogen peroxide solutions, finding that POM is more susceptible to oxidative degradation due to $\hbox {H}_{2}\hbox {O}_{2}$ infiltration^32^. This research underscores understanding polymer behavior under oxidative conditions for harsh environment applications. These studies illustrate ReaxFF MD’s versatility in addressing both mechanical reinforcement and chemical degradation, providing atomic-level insights that complement experimental research.

Despite the growing interest in supercritical water gasification as a sustainable method for plastic recycling, there remains a critical gap in understanding the molecular-level mechanisms governing this process, particularly for POM. This study is designed to address this knowledge gap by thoroughly investigating the intricate decomposition pathways and reaction kinetics involved in POM’s transformation during SCWG. Through detailed examination of the interactions between POM and supercritical water, this research aims to uncover the fundamental mechanisms that drive the gasification process. Understanding these molecular-level insights is not only essential for optimizing the efficiency of SCWG but also pivotal for enhancing product yields and potentially revolutionizing plastic recycling technologies. By identifying key reaction pathways and controlling factors, this study seeks to provide a foundation for developing customized processes tailored to maximize resource recovery from plastic waste, thereby contributing to broader efforts in sustainable materials management.

## Models and methods

In molecular dynamics (MD) simulations, employing model compounds is an essential strategy for simplifying complex real-world systems into computationally tractable representations. These models, though simplified in structure and computational requirements, are meticulously designed to preserve the critical structural and dynamic features of the material being studied. For this investigation, we selected a model represented by the formula $\hbox {C}_{40}\hbox {H}_{82}\hbox {O}_{40}$ (consists of 40 monomers) to depict polyoxymethylene (POM). This model strikes an optimal balance between accurately capturing POM’s behavior and managing computational resources effectively. Our simulation system was composed of 5 such POM molecules, chosen to reflect a realistic yet computationally feasible representation.

**Fig. 1:**
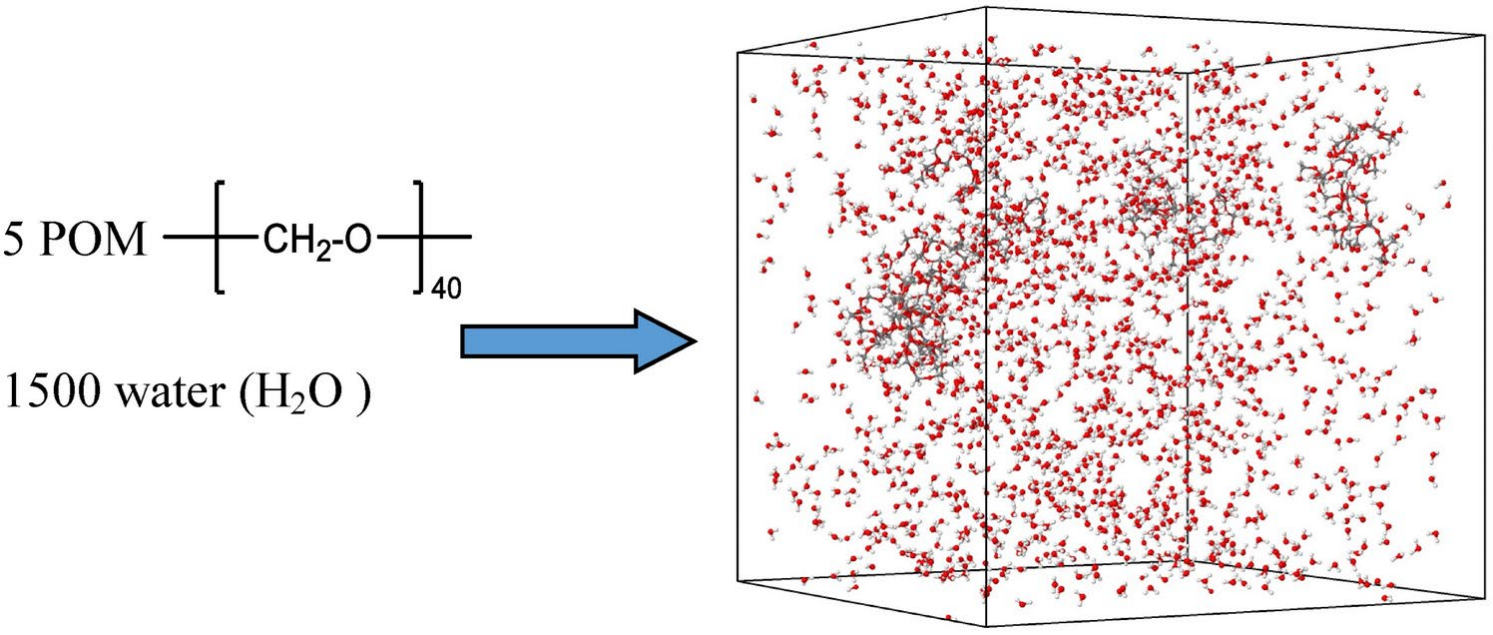
Structural representation of POM (polyoxymethylene) and System 4 containing 5 POM molecules and 1500 water molecules, illustrating the system setup for the SCGW process. Hydrogen is depicted in white, while carbon and oxygen are shown in gray and red, respectively.

To explore the impact of water content on the SCWG process, we conducted a series of simulations where water concentration within the simulation box was systematically varied from 60% to 82%. This range mirrors typical moisture levels encountered in SCWG processes under extreme conditions, allowing us to examine how varying degrees of hydration influence degradation dynamics. The overall density of our simulated system was maintained at approximately 0.2 g/cm$^3$, aligning with the gasification process simulations previously reported by Pang et al.^38^. This consistency ensures comparability and reinforces the validity of our findings within the broader context of SCWG research. Simulation parameters and configurations are summarized in Table 1, providing a clear overview of each case study. An illustrative snapshot of system 4 is presented in Fig. 1, offering a visual representation of the system’s structure under these conditions.

**Table 1:** Model systems for SCWG simulation of POM in ReaxFF MD.

System	POM (molecules)	Water (molecules)	No. of atoms	Box length (Å)
1	5 (40%)	500 (60%)	2310	50.00
2	5 (27%)	900 (73%)	3510	57.03
3	5 (23%)	1100 (77%)	4110	60.00
4	5 (18%)	1500 (82%)	5310	65.09

The number of molecules of POM and water in each system are listed. The value in parentheses (%) show the mass fraction percentage of POM and water.

### ReaxFF molecular dynamics and simulation method

The ReaxFF (reactive force field) method was employed for this study due to its ability to accurately describe molecular interactions, particularly chemical reactions. Developed by Adri van Duin and William A. Goddard III, ReaxFF combines molecular mechanics and dynamics with a reactive potential energy function, enabling the modeling of bond breaking and formation^36^. ReaxFF’s ability to adjust bond lengths and angles in response to changing chemical environments makes it a powerful tool for studying molecular-level properties and reactions. For this method, the total energy of a system is expressed in Eq. (1) as follows:

1 $$\begin{aligned} E_{\text {system }}=E_{\text {bond }}+E_{\text {over }}+E_{\text {under }}+E_{\text {val }}+E_{\text {pen }}+ E_{\text {tors }}+E_{\text {conj }}+E_{\text {vdWaals }}+E_{\text {Coulomb }} \end{aligned}$$

where $E_{\text {system }}$ represents the total energy of the system, and $E_{\text {bond }}$, $E_{\text {over }}$, $E_{\text {under }}$, $E_{\text {val }}$, $E_{\text {pen }}$, $E_{\text {tors }}$, $E_{\text {conj }}$, $E_{\text {vdWaals }}$, and $E_{\text {Coulomb }}$ represent the bond energy based on the bond order, the over-coordination atom energy, the under-coordination atom energy, the valence angle energy, the penalty energy for the double bond, the torsion angle energy, conjugated effect energies, and the van der Waals and Coulomb forces for non-bonding interactions, respectively. For example, the terms $E_{\text {over}}$ and $E_{\text {under}}$ in the ReaxFF force field address deviations from ideal atomic coordination, ensuring realistic bonding environments in simulations. $E_{\text {over}}$ represents the energy penalty for over-coordinated atoms, which occur when an atom forms more bonds than its typical valence allows. This term discourages unrealistic over-bonding, such as a carbon atom forming five bonds instead of its usual four. On the other hand, $E_{\text {under}}$ penalizes under-coordinated atoms, which have fewer bonds than their valence would typically permit, as seen in radicals or fragmented systems. By incorporating these terms, ReaxFF ensures that atoms maintain chemically reasonable coordination numbers, guiding the system toward stable and physically accurate configurations.

Unlike traditional force fields, which rely on fixed bonding arrangements, ReaxFF employs a bond-order formalism that dynamically updates bonding interactions during simulations. This allows it to describe bond formation, bond breaking, and changes in molecular structure in response to varying chemical environments. The fundamental strength of ReaxFF lies in its ability to simulate reactive processes^36^. At the core of ReaxFF is the concept of bond order, which quantifies the strength and type of bonds between atoms. The bond order is calculated based on interatomic distances and is continuously updated during simulations, enabling the force field to adapt to changes in the system. For further details, readers are encouraged to refer to the original ReaxFF paper^36^.

In this research, we utilized the potential function parameters developed by Vashisth et al.^39^. These parameters have been thoroughly validated using experimental data and density functional theory (DFT) calculations, establishing their reliability for studying organic molecular reactions. The ReaxFF simulations were conducted using Large-scale Atomic/Molecular Massively Parallel Simulator (LAMMPS)^40^, a widely-used classical molecular dynamics software designed for materials modeling. To ensure the reliability of our simulations, we followed well-established protocols for system setup, equilibration, and production runs^30,41–45^. Our systems were initialized with random configurations and underwent a series of energy minimization and equilibration steps to achieve a stable state prior to the production phase. Each system was subjected to a 5 picosecond (ps) relaxation period at 300 K in the NVT ensemble (constant number of particles N, volume V, and temperature T), ensuring energy minimization before proceeding with further simulations.This systematic approach ensures that our molecular dynamics simulations are both accurate and reproducible (see more details in the SI), providing a solid foundation for studying the behavior of organic molecules under various conditions.

To enhance the efficiency of chemical reactions and address the time and scale limitations inherent in conventional experimental techniques, our simulations were conducted at elevated temperatures exceeding those typically observed in experiments. This approach has been validated in prior studies for accelerating thermal processes in various organic materials^41–45^. In this work, temperatures were set within the range of 1400 K to 3100 K. This range is lower than experimental conditions reported by Lu et al.^29^, who studied SCWG at temperatures between 700 K and 1000 K. The reason for this discrepancy lies in the nature of MD simulations, which are computationally intensive and limited by their timescale. To ensure sufficient reaction occurrences within the MD simulation timeframe, it is common to simulate reactions at higher temperatures than experimental settings^41–45^. The heating rate was maintained at 20 K per picosecond (ps), aligning with methodologies employed in previous ReaxFF studies^44^. Additionally, a time step of 0.25 femtoseconds (fs) was utilized, ensuring precise capture of system dynamics while keeping computational demands manageable^41–44^. This strategy not only accelerates the reaction kinetics but also allows for a more thorough investigation of the underlying mechanisms, providing valuable insights into organic material transformations under extreme conditions. A comprehensive table outlining the crucial simulation parameters utilizing the ReaxFF force field can be found in the Supplementary Information.

## Results and discussion

### Reaction mechanism

The SCWG of POM involves a intricate series of chemical transformations that ultimately yield a variety of gaseous products. In this study, molecular dynamics (MD) simulations are employed to elucidate the complex reaction mechanisms underlying the SCWG of POM. These simulations provide valuable insights into the SCWG pathways across a broad thermal spectrum, ranging from 1400 K to 3100 K. The HTG process refers to the decomposition and conversion of polymers into gaseous products under elevated temperatures, which is significantly influenced by temperature variations. To visualize the progression of the system under different conditions, we present a series of snapshot images that capture the POM decomposition process at various simulation times.

**Fig. 2:**
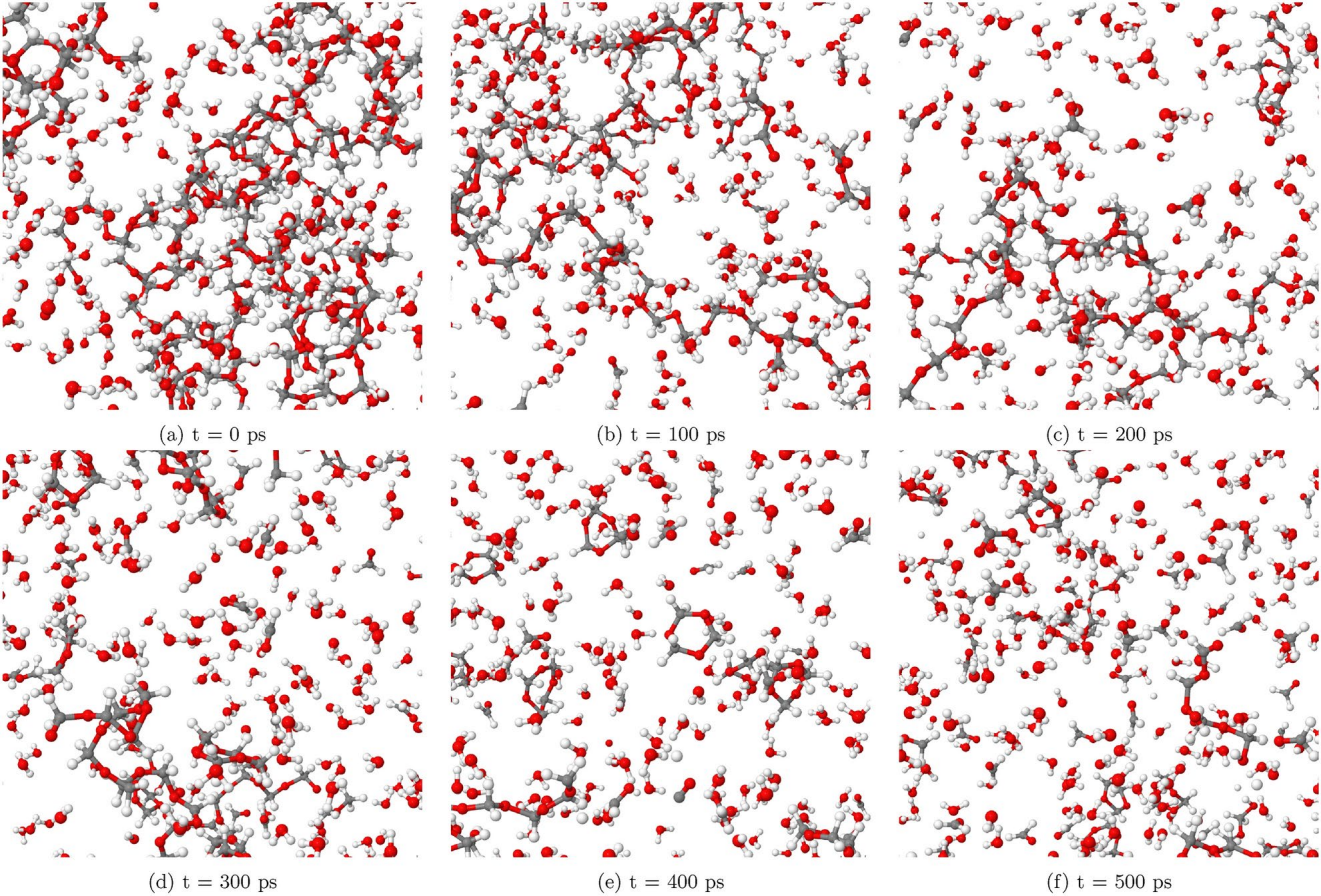
Selected snapshots of the SCWG process of POM (system 1) at T = 1400K at different simulation times, illustrating limited gas product formation at this temperature.

At a lower temperature of T = 1400 K, as illustrated in Fig. 2, the SCWG process demonstrates reduced efficiency compared to higher temperatures. POM undergoes fragmentation, breaking down into smaller molecular pieces. However, the resulting carbon chains remain relatively large, spanning from C5 to C20 in length. This indicates that the polymer’s degradation is incomplete at this temperature. The production of gaseous products such as hydrogen and CO is notably limited by 500 picoseconds (ps), suggesting that the reaction conditions are not conducive for robust gasification at T = 1400 K. This inefficiency underscores the importance of optimizing temperature parameters to achieve effective SCWG.

**Fig. 3:**
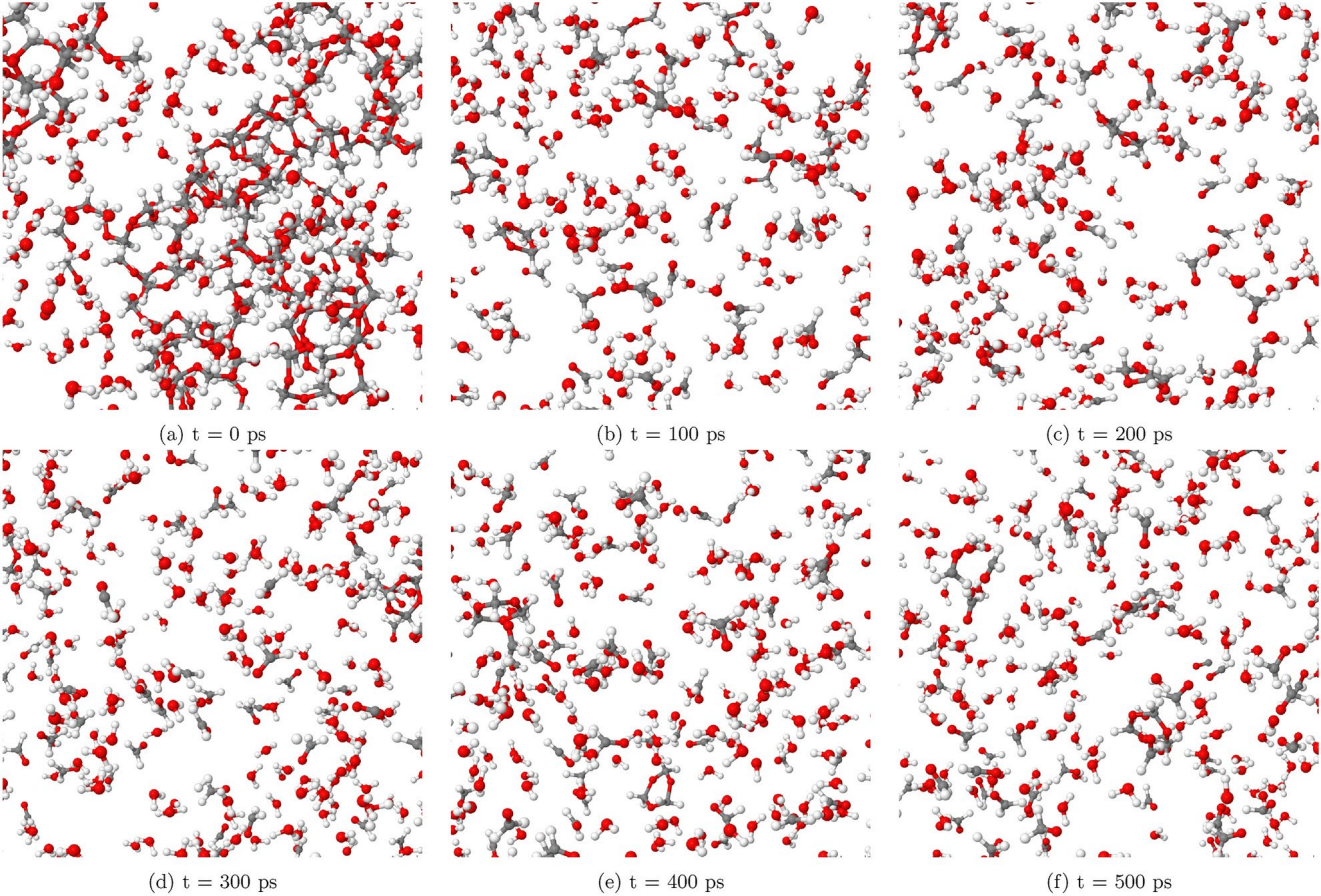
Selected snapshots of the SCWG process of POM (system 1) at T = 1700K at different simulation times, illustrating the enhanced gas product formation.

Figure 3 illustrates the transformation of POM at T = 1700 K within a system containing 60% water by weight. These snapshots reveal the breakdown of POM molecules into smaller hydrocarbon chains and gases. Notably, the POM polymer is observed to completely transform into small fragments after 100 picoseconds (ps). By the end of the simulation at 500 ps, a significant concentration of hydrogen and CO gases is evident.

We have observed that at T = 1400 K, the reaction is limited in our simulations, while it occurs at T = 1700 K. In contrast, experimental data demonstrates reactions taking place at much lower temperatures^29^. This discrepancy can be attributed to the differences in time scales between simulations and experiments. Our simulations are conducted on a nanosecond scale, whereas experimental data is obtained over minutes or hours^29^. Due to the very short simulation time, higher temperatures are required for reactions to occur within the limited timeframe of our simulations. As a result, the apparent temperature threshold for reactions in our study may be higher than what would be observed in actual experiments with longer reaction times. It is essential to consider these factors when comparing simulation results with experimental data and interpreting the implications for real-world applications.

Analyzing representative elementary reactions (RERs) is fundamental to understanding the intricate mechanisms of SCWG. These reactions provide essential insights into the fundamental steps involved in polymer decomposition, particularly for POM, a widely used plastic polymer. At temperatures exceeding 1400 K, the SCWG process becomes significantly more active, with numerous reactions occurring to facilitate the breakdown of POM. At T = 1500 K, the initial stages of POM decomposition involve several key elementary reactions that are critical for transforming the polymer into smaller fragments. These reactions are cataloged in Table 2, which highlights the specific pathways and products associated with the degradation process at this temperature.

**Table 2:** Representative elementary reactions of POM ($\hbox {C}_{40}\hbox {H}_{82}\hbox {O}_{40}$) in the SCWG process obtained by ReaxFF simulations at T = 1500 K.

Nr.	Reactions
1	$\hbox {C}_{18}\hbox {H}_{38}\hbox {O}_{18}+ \hbox {C}_{22}\hbox {H}_{45}\hbox {O}_{22}^{\cdot }\rightarrow \hbox {C}_{40}\hbox {H}_{82}\hbox {O}_{40}+\hbox {H}^{\cdot }$
2	$\hbox {C}_{40}\hbox {H}_{82}\hbox {O}_{40} + \hbox {CH}_{3}\hbox {O}^{\cdot }\rightarrow \hbox {CH}_{4}\hbox {O}+ \hbox {C}_{40}\hbox {H}_{81}\hbox {O}_{40}^{\cdot }$
3	$\hbox {C}_{40}\hbox {H}_{82}\hbox {O}_{40} +\hbox {H}^{\cdot }\rightarrow \hbox {C}_{16}\hbox {H}_{34}\hbox {O}_{16}+ \hbox {C}_{24}\hbox {H}_{49}\hbox {O}_{24}^{\cdot }$
4	$\hbox {C}_{40}\hbox {H}_{82}\hbox {O}_{40} + \hbox {H}^{\cdot }\rightarrow \hbox {C}_{37}\hbox {H}_{75}\hbox {O}_{36}^{\cdot }+ \hbox {C}_{3}\hbox {H}_{8}\hbox {O}_{4}$
5	$\hbox {C}_{40}\hbox {H}_{82}\hbox {O}_{40} +\hbox {H}^{\cdot }\rightarrow \hbox {C}_{18}\hbox {H}_{37}\hbox {O}_{17}^{\cdot } + \hbox {C}_{22}\hbox {H}_{46}\hbox {O}_{23}$
6	$\hbox {C}_{40}\hbox {H}_{82}\hbox {O}_{40}\rightarrow \hbox {C}_{28}\hbox {H}_{57}\hbox {O}_27^{\cdot }+\hbox {C}_{12}\hbox {H}_{25}\hbox {O}_{13}^{\cdot }$
7	$\hbox {C}_{40}\hbox {H}_{82}\hbox {O}_{40}\rightarrow \hbox {C}_{27}\hbox {H}_{55}\hbox {O}_{27}^{\cdot }+\hbox {C}_{13}\hbox {H}_{27}\hbox {O}_{13}^{\cdot }$
8	$\hbox {C}_{40}\hbox {H}_{82}\hbox {O}_{40}\rightarrow \hbox {C}_{21}\hbox {H}_{43}\hbox {O}_{21}^{\cdot }+ \hbox {C}_{19}\hbox {H}_{39}\hbox {O}_{19}^{\cdot }$
9	$\hbox {C}_{40}\hbox {H}_{82}\hbox {O}_{40}\rightarrow \hbox {C}_{18}\hbox {H}_{37}\hbox {O}_{18}^{\cdot }+ \hbox {C}_{22}\hbox {H}_{45}\hbox {O}_{22}^{\cdot }$
10	$\hbox {C}_{40}\hbox {H}_{82}\hbox {O}_{40}\rightarrow \hbox {C}_{8}\hbox {H}_{17}\hbox {O}_{7}^{\cdot }+ \hbox {C}_{32}\hbox {H}_{65}\hbox {O}_{33}^{\cdot }$
11	$\hbox {C}_{40}\hbox {H}_{82}\hbox {O}_{40}\rightarrow \hbox {C}_{7}\hbox {H}_{15}\hbox {O}_{7}^{\cdot }+ \hbox {C}_{33}\hbox {H}_{67}\hbox {O}_{33}^{\cdot }$
12	$\hbox {C}_{40}\hbox {H}_{82}\hbox {O}_{40}\rightarrow \hbox {C}_{5}\hbox {H}_{11}\hbox {O}_{4}^{\cdot }+ \hbox {C}_{35}\hbox {H}_{71}\hbox {O}_{36}^{\cdot }$
13	$\hbox {C}_{37}\hbox {H}_{75}\hbox {O}_{37}^{\cdot }+ \hbox {C}_{3}\hbox {H}_{7}\hbox {O}_{3}^{\cdot } \rightarrow \hbox {C}_{40}\hbox {H}_{82}\hbox {O}_{40}$
14	$\hbox {C}_{35}\hbox {H}_{71}\hbox {O}_{34}^{\cdot } + \hbox {C}_{5}\hbox {H}_{11}\hbox {O}_{6}^{\cdot }\rightarrow \hbox {C}_{40}\hbox {H}_{82}\hbox {O}_{40}$
15	$\hbox {C}_{22}\hbox {H}_{45}\hbox {O}_{21}^{\cdot }+ \hbox {C}_{18}\hbox {H}_{37}\hbox {O}_{19}^{\cdot }\rightarrow \hbox {C}_{40}\hbox {H}_{82}\hbox {O}_{40}$
16	$\hbox {C}_{15}\hbox {H}_{31}\hbox {O}_{14}^{\cdot } + \hbox {C}_{25}\hbox {H}_{51}\hbox {O}_{26}^{\cdot }\rightarrow \hbox {C}_{40}\hbox {H}_{82}\hbox {O}_{40}$
17	$\hbox {C}_{7}\hbox {H}_{15}\hbox {O}_{6}^{\cdot } +\hbox {C}_{33}\hbox {H}_{67}\hbox {O}_{34}^{\cdot } \rightarrow \hbox {C}_{40}\hbox {H}_{82}\hbox {O}_{40}$

Table 2 presents a comprehensive list of representative elementary reactions (RERs) for polyoxymethylene (POM, $\hbox {C}_{40}\hbox {H}_{82}\hbox {O}_{40}$) undergoing SCWG at T = 1500 K. These RERs are derived from ReaxFF molecular dynamics simulations and offer valuable insights into the fundamental mechanisms of POM degradation under SCWG conditions. The reactions listed include both decomposition and recombination processes, highlighting the dynamic nature of the SCWG process. Decomposition reactions involve the cleavage of POM into smaller fragments, often accompanied by the release of hydrogen radicals ($\hbox {H}^{\cdot }$). For instance, Reaction 3 shows POM breaking down into $\hbox {C}_{16}\hbox {H}_{34}\hbox {O}_{16}$ and a radical species $\hbox {C}_{24}\hbox {H}_{49}\hbox {O}_{24}^{\cdot }$, demonstrating how the polymer chain cleaves at specific points. Similarly, Reaction 6 splits POM into $\hbox {C}_{28}\hbox {H}_{57}\hbox {O}_27^{\cdot }$ and $\hbox {C}_{12}\hbox {H}_{25}\hbox {O}_{13}^{\cdot }$, indicating cleavage points that result in both medium-sized and small fragments. Recombination reactions, such as Reaction 13, demonstrate how smaller fragments can recombine to reform the original polymer or similar species. This bidirectional nature suggests a dynamic equilibrium where decomposition and recombination pathways coexist. The presence of radicals (denoted by the dot) underscores the free-radical mechanism typical of SCWG processes, where radicals act as both intermediates and catalysts for further reactions. The fragmentation patterns reveal that POM breaks down into a diverse range of smaller fragments, varying in size and composition. For example, Reaction 10 produces a small fragment ($\hbox {C}_{8}\hbox {H}_{17}\hbox {O}_{7}^{\cdot }$) and a larger residue ($\hbox {C}_{32}\hbox {H}_{65}\hbox {O}_{33}^{\cdot }$). This diversity highlights the non-uniform cleavage points along the polymer chain. Formation of small organic molecule is another critical aspect of SCWG, as seen in Reaction 2, where POM reacts with $\hbox {CH}_{3}\hbox {O}^{\cdot }$ to produce methanol ($\hbox {CH}_{4}\hbox {O}$) and a radical species.

**Table 3:** Representative elementary reactions of water and gases in the SCWG process obtained by ReaxFF simulations at T = 1500 K.

Nr.	Reactions
1	$\hbox {CH}_{3}\hbox {O}^{\cdot }\rightarrow \hbox {CH}_{2}\hbox {O} + \hbox {H}^{\cdot }$
2	$\hbox {C}_{3}\hbox {H}_{7}\hbox {O}_{3}^{\cdot } \rightarrow + \hbox {C}_{3}\hbox {H}_{6}\hbox {O}_{3}+ \hbox {H}^{\cdot }$
3	$\hbox {C}_{2}\hbox {H}_{5}\hbox {O}_{2}^{\cdot }\rightarrow \hbox {CH}_{2}\hbox {O}+ \hbox {CH}_{3}\hbox {O}^{\cdot }$
4	$\hbox {C}_{4}\hbox {H}_{9}\hbox {O}_{4}^{\cdot } \rightarrow \hbox {CH}_{2}\hbox {O} + \hbox {C}_{3}\hbox {H}_{7}\hbox {O}_{3}^{\cdot }$
5	$\hbox {C}_{11}\hbox {H}_{23}\hbox {O}_{11}^{\cdot } \rightarrow \hbox {CH}_{2}\hbox {O}+ \hbox {C}_{10}\hbox {H}_{21}\hbox {O}_{10}^{\cdot }$
6	$\hbox {C}_{12}\hbox {H}_{25}\hbox {O}_{12}^{\cdot } + \hbox {C}_{4}\hbox {H}_{8}\hbox {O}_{3}\rightarrow \hbox {C}_{6}\hbox {H}_{13}\hbox {O}_{6}^{\cdot } + \hbox {C}_{10}\hbox {H}_{20}\hbox {O}_{9}$
7	$\hbox {C}_{14}\hbox {H}_{29}\hbox {O}_{14}^{\cdot } \rightarrow \hbox {C}_{3}\hbox {H}_{6}\hbox {O}_{3} + \hbox {C}_{11}\hbox {H}_{23}\hbox {O}_{11}^{\cdot }$
8	$\hbox {C}_{5}\hbox {H}_{11}\hbox {O}_{5}^{\cdot }\rightarrow \hbox {C}_{3}\hbox {H}_{6}\hbox {O}_{3} +\hbox {C}_{2}\hbox {H}_{5}\hbox {O}_{2}^{\cdot }$
9	$\hbox {C}_{22}\hbox {H}_{45}\hbox {O}_{22}^{\cdot }\rightarrow \hbox {C}_{3}\hbox {H}_{6}\hbox {O}_{3}+ \hbox {C}_{19}\hbox {H}_{39}\hbox {O}_{19}^{\cdot }$

Table 3 illustrates elementary reactions involving water and gases during SCWG at T = 1500 K. These reactions primarily consist of decomposition processes where larger organic molecules break down into smaller fragments, releasing gaseous products such as formaldehyde ($\hbox {CH}_{2}\hbox {O}$) and hydrogen radicals ($\hbox {H}^{\cdot }$). For instance, reactions like $\hbox {CH}_{3}\hbox {O}^{\cdot }\rightarrow \hbox {CH}_{2}\hbox {O} + \hbox {H}^{\cdot }$ and $\hbox {C}_{4}\hbox {H}_{9}\hbox {O}_{4}^{\cdot } \rightarrow \hbox {CH}_{2}\hbox {O} + \hbox {C}_{3}\hbox {H}_{7}\hbox {O}_{3}^{\cdot }$ demonstrate the cleavage of methyl groups with oxygen and more complex structures, respectively. Notably, Reaction 6 stands out as a combination reaction where $\hbox {C}_{12}\hbox {H}_{25}\hbox {O}_{12}^{\cdot }$ reacts with $\hbox {C}_{4}\hbox {H}_{8}\hbox {O}_{3}$ to form smaller fragments ($\hbox {C}_{6}\hbox {H}_{13}\hbox {O}_{6}^{\cdot }$ and $\hbox {C}_{10}\hbox {H}_{20}\hbox {O}_{9}$), indicating some recombination processes alongside decomposition. The presence of radicals, such as $\hbox {CH}_{3}\hbox {O}^{\cdot }$, underscores the free-radical mechanisms driving these reactions, typical in high-temperature gasification. Additionally, even very large molecules like $\hbox {C}_{22}\hbox {H}_{45}\hbox {O}_{22}^{\cdot }$ break down efficiently into smaller fragments ($\hbox {C}_{3}\hbox {H}_{6}\hbox {O}_{3}$ and $\hbox {C}_{19}\hbox {H}_{39}\hbox {O}_{19}^{\cdot }$), highlighting the process’s effectiveness in handling complex organic structures. Overall, this table provides valuable insights into the dynamic interplay of decomposition and combination reactions in SCWG, contributing to the conversion of plastic waste into reusable gases.

**Table 4:** Representative elementary reactions of water and gases in the SCWG process obtained by ReaxFF simulations at T = 2700K.

Nr.	Reactions
1	$\hbox {H}_{2}\hbox {O} + \hbox {H}^{\cdot }\rightarrow \hbox {H}_{2} + \hbox {HO}^{\cdot }$
2	$\hbox {CH}_{2}\hbox {O} + \hbox {HO}^{\cdot }\rightarrow \hbox {H}_{2}\hbox {O} + \hbox {CHO}^{\cdot }$
3	$\hbox {H}_{2}\hbox {O} + \hbox {H}^{\cdot }\rightarrow \hbox {H}_{2} + \hbox {HO}^{\cdot }$
4	$\hbox {CH}_{2}\hbox {O}\rightarrow \hbox {CHO}^{\cdot }+ \hbox {H}^{\cdot }$
5	$\hbox {H}^{\cdot } + \hbox {H}^{\cdot } \rightarrow \hbox {H}_{2}$
6	$\hbox {CHO}^{\cdot } \rightarrow \hbox {CO} + \hbox {H}^{\cdot }$
7	$\hbox {CHO}_{2}^{\cdot }\rightarrow \hbox {CO}_{2} + \hbox {H}^{\cdot }$
8	$\hbox {CO} + \hbox {HO}^{\cdot } \rightarrow \hbox {CHO}_{2}^{\cdot }$
9	$\hbox {CO}_{2} + \hbox {CHO}^{\cdot } \rightarrow \hbox {CHO}_{2}^{\cdot } + \hbox {CO}$
10	$\hbox {CO}_{2} + \hbox {H}^{\cdot } \rightarrow \hbox {CHO}_{2}^{\cdot }$

Table 4 illustrates elementary reactions occurring during SCWG at T = 2700 K. At this elevated temperature, the gasification process is significantly more complete compared to lower temperatures, such as T = 1500 K. The higher thermal energy at 2700 K facilitates the breaking of stronger chemical bonds, leading to a greater extent of decomposition of organic molecules into simpler gaseous products like carbon monoxide (CO), carbon dioxide ($\hbox {CO}_{2}$), and molecular $\hbox {H}_{2}$.

For instance, reactions involving the breakdown of formaldehyde ($\hbox {CH}_{2}\hbox {O}$) into CO and $\hbox {H}^{\cdot }$ (as seen in Reaction 4 and 6 of the Table 4 ) are more pronounced at this temperature, contributing to increased CO production. Additionally, interactions between radicals such as $\hbox {CHO}^{\cdot }$ and $\hbox {HO}^{\cdot }$ enhance the formation of $\hbox {CO}_{2}$ through pathways like Reaction 7, where $\hbox {CHO}_{2}^{\cdot }$ decomposes into $\hbox {CO}_{2}$ and $\hbox {H}^{\cdot }$. The elevated temperature also promotes the combination of hydrogen radicals ($\hbox {H}^{\cdot }$) to form molecular hydrogen, as shown in Reaction 5. This increase in $\hbox {H}_{2}$ production is crucial for enhancing the overall efficiency of SCWG, as it contributes to a higher yield of reusable gases. These observations underscore the critical role of temperature in SCWG processes. Higher temperatures not only accelerate reaction rates but also shift equilibrium positions and influence product selectivity, thereby optimizing gasification outcomes. By understanding how temperature affects these elementary reactions, researchers can develop more efficient strategies for converting plastic waste into valuable gaseous products, advancing sustainable waste management practices.

### Product formation at different temperatures

**Fig. 4:**
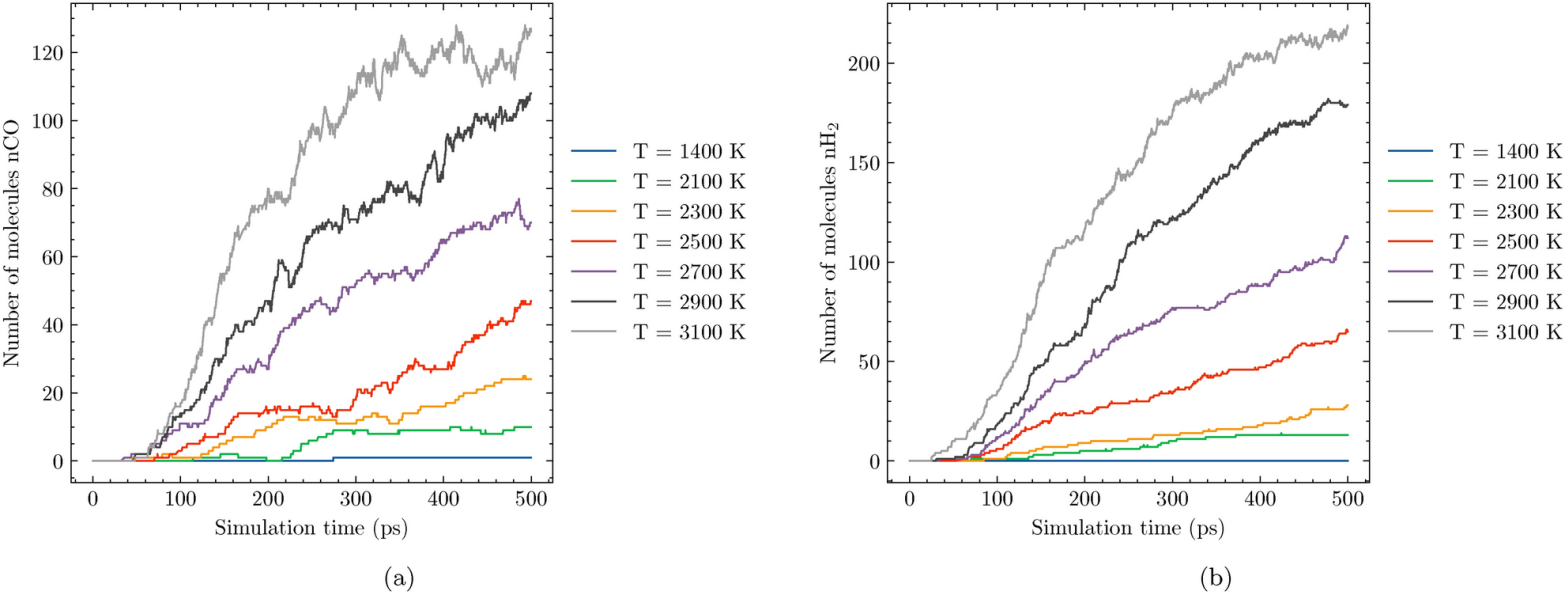
Evolution of CO (**a**) and $\hbox {H}_{2}$ (**b**) molecules during the SCWG process in System 1 at different temperatures.

In this section, we examine how temperature influences the formation of gaseous products during SCWG. Figure 4 illustrates the evolution of carbon monoxide (CO) and molecular $\hbox {H}_{2}$ concentrations over a 500 picosecond (ps) simulation period across a temperature range of 1400 K to 3100 K. The results reveal that at lower temperatures, such as T = 1400 K, the formation of CO and $\hbox {H}_{2}$ is significantly limited. As temperature increases, there is a noticeable rise in both CO and $\hbox {H}_{2}$ concentrations, with their yields becoming more substantial at higher thermal conditions. This trend underscores the importance of temperature in driving the gasification process, as increased thermal energy facilitates the breaking of chemical bonds and enhances reaction kinetics.

Notably, across all simulated temperatures, molecular $\hbox {H}_{2}$ is produced in greater quantities compared to CO. This can be attributed to the specific reaction pathways active under SCWG conditions. For instance, the formation of $\hbox {H}_{2}$ is often favored due to its simpler molecular structure and lower activation energy requirements for synthesis. This analysis demonstrates that increasing temperature significantly enhances the formation of CO and $\hbox {H}_{2}$, with hydrogen consistently outperforming carbon monoxide in terms of production volume. These insights highlight the critical role of thermal regulation in achieving optimal outcomes for SCWG processes.

To provide an in-depth understanding of the POM cleavage process, the evolution of molecular fragments at various temperatures is illustrated. In the Figs. S2 and S3 of the Supplementary Information, we present the largest molecular mass and its average for each temperature considered. Our findings reveal that as the temperature increases, the average molecular mass decreases significantly, suggesting enhanced fragmentation and decomposition of POM. This demonstrates how higher temperatures facilitate more efficient cleavage and breakdown of polymer chains in the supercritical water gasification process.

**Fig. 5:**
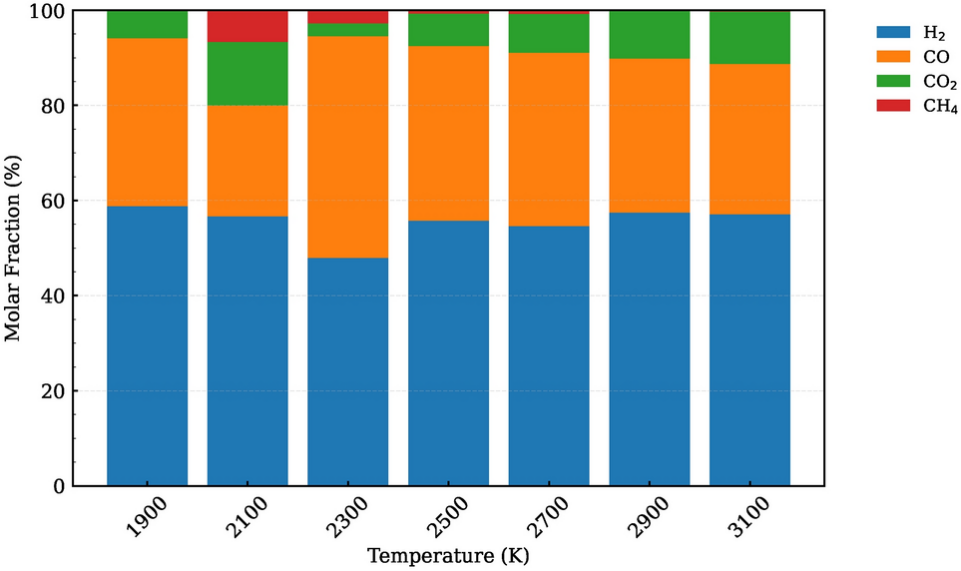
Gas molar fraction of the HTG process for system 1 at different temperatures.

Figure 5 presents the molar fractions of gaseous products resulting from the SCWG of POM. The primary gaseous products identified are molecular $\hbox {H}_{2}$, CO, $\hbox {CO}_{2}$, and a negligible amount of methane ($\hbox {CH}_{4}$). These gaseous products can be explained by the following main reactions.

Steam reforming reaction of plastics:

2 $$\begin{aligned} \hbox {POM} + \hbox {H}_{2}\hbox {O} \longrightarrow \hbox {CO} + \hbox {CO}_{2} + \hbox {H}_{2} \end{aligned}$$

Water gas shift reaction:

3 $$\begin{aligned} \hbox {CO} + \hbox {H}_{2}\hbox {O} \longrightarrow \hbox {CO}_{2} + \hbox {H}_{2} \end{aligned}$$

Methanation reaction:

4 $$\begin{aligned} \hbox {CO} + 3\hbox {H}_{2} \longrightarrow \hbox {CH}_{4} + \hbox {H}_{2}\hbox {O} \end{aligned}$$

Methane decomposition at high temperature:

5 $$\begin{aligned} \hbox {CH}_{4} + \hbox {CO}_{2} \longrightarrow 2\hbox {CO} + 2\hbox {H}_{2} \end{aligned}$$

The molar fraction distribution reveals that $\hbox {H}_{2}$ is the dominant gas, accounting for approximately 60% of the total gaseous products. This is followed by CO, which constitutes around 30%, while $\hbox {CO}_{2}$ contributes less than 10%. The minimal presence of $\hbox {CH}_{4}$ (barely detectable in most cases) is intriguing and suggests that its formation is highly temperature-dependent.

When comparing the gaseous product distribution at temperatures of 2300 K and 2100 K, we observe significant differences in the levels of CO and $\hbox {CO}_{2}$. At higher temperatures (e.g., 2300 K), reactions are more complete, resulting in a substantial increase in CO levels compared to lower temperatures (e.g., 2100 K). At 2100 K, the formation of $\hbox {CH}_{4}$ may occur through the methanation reaction. However, at these higher temperatures, methane decomposes via the following reaction. Consequently, at higher temperatures such as 2300 K – 2700 K, the levels of methane decrease due to its decomposition. Therefore, at elevated temperatures, there is no significant methane present in the gaseous product mixture. At higher temperatures, specifically at 2900 K and 3100 K, the gasification process becomes more complete. Under these conditions, the reaction conditions are less conducive to methane formation, resulting in its near absence from the gaseous products. This observation aligns with the understanding that elevated thermal energy promotes more thorough decomposition of organic matter, favoring the production of simpler gases like $\hbox {H}_{2}$ and CO over complex hydrocarbons such as $\hbox {CH}_{4}$.

At high temperature range of 2300–3100 K, the increased fraction of $\hbox {CO}_{2}$ in the gaseous product mixture can be attributed to the enhanced favorability of the water gas shift reaction. At higher temperatures, this endothermic reaction proceeds more efficiently, resulting in an increased conversion of carbon monoxide and water into carbon dioxide and hydrogen.

The dominance of $\hbox {H}_{2}$ and CO can be attributed to their lower activation energy requirements for formation under SCWG conditions. Additionally, the high reactivity of hydrogen radicals at elevated temperatures facilitates their combination into stable molecular $\hbox {H}_{2}$, contributing significantly to its molar fraction. These findings underscore the critical role of temperature in dictating product distribution during SCWG. By optimizing thermal conditions, researchers can enhance the selectivity toward desirable gases like $\hbox {H}_{2}$ and CO, while minimizing unwanted byproducts such as $\hbox {CH}_{4}$. This has important implications for improving the efficiency and sustainability of plastic-to-gas conversion processes in industrial applications.

When comparing our simulation results with the experimental findings reported by Lu et al.^29^, we observe an excellent agreement in terms of gas composition. Both sets of data consistently show a dominance of molecular $\hbox {H}_{2}$, followed by CO, and $\hbox {CO}_{2}$ with the least contribution. Furthermore, both our simulations and their experiments indicate only minimal traces of methane ($\hbox {CH}_{4}$) and the absence of C2 gases, such as ethylene or ethane. This close alignment in observed gas species underscores the validity of our computational approach.

A particularly notable aspect of this comparison is the consistent trend observed in the molar fraction of $\hbox {H}_{2}$. In both the experimental data and our simulations, there is a clear increase in the proportion of $\hbox {H}_{2}$ as temperature rises. This identical trend provides strong evidence that our ReaxFF simulation accurately captures the thermodynamic and kinetic behavior of the gasification process.

The agreement between our simulated results and the experimental findings by Lu et al.^29^ not only validates the predictive capabilities of our computational model but also reinforces its utility for studying SCWG processes under various conditions. Such consistency is crucial for gaining confidence in simulation-based approaches, enabling them to serve as valuable tools for exploring scenarios that may be difficult or costly to investigate experimentally.

**Fig. 6:**
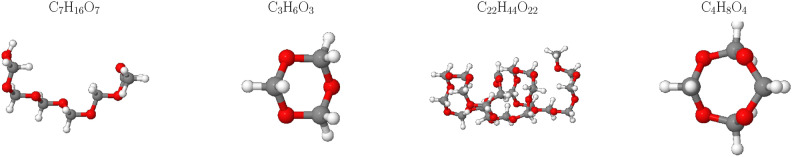
Snapshot of the largest product molecules with their chemical formula during the SCWG process of system 1 at temperature at 1400 K. The gray and white color represent for C, and H atoms, respectively.

The impact of temperature on product formation during SCWG of POM was thoroughly investigated. At a lower temperature of T = 1400 K, as depicted in Fig. 6, the decomposition process is notably slow and incomplete. This results in the persistence of large organic fragments such as $\hbox {C}_{22}\hbox {H}_{44}\hbox {O}_{22}$ and $\hbox {C}_{7}\hbox {H}_{16}\hbox {O}_{7}$, alongside smaller organic compounds like $\hbox {C}_{3}\hbox {H}_{6}\hbox {O}_{3}$. The presence of these larger fragments indicates that insufficient thermal energy is available to fully break down the polymer into simpler components.

In contrast, at higher temperatures (e.g., T = 2700 K), the decomposition becomes significantly more efficient. At such elevated temperatures, the reaction process is complete, with no large organic fragments remaining. This drastic improvement in decomposition efficiency can be attributed to the increased thermal energy available at higher temperatures, which facilitates the breaking of stronger chemical bonds and promotes more thorough fragmentation of the polymer. The transition from incomplete to complete decomposition underscores the critical role of temperature in SCWG processes. At lower temperatures, insufficient energy hinders the breakdown of larger molecular structures, leading to residual organic fragments. As temperature increases, the provision of adequate thermal energy enables the reaction to proceed to completion, resulting in a higher yield of gaseous products and no significant retention of large organic compounds.

### Effect of water content

**Fig. 7:**
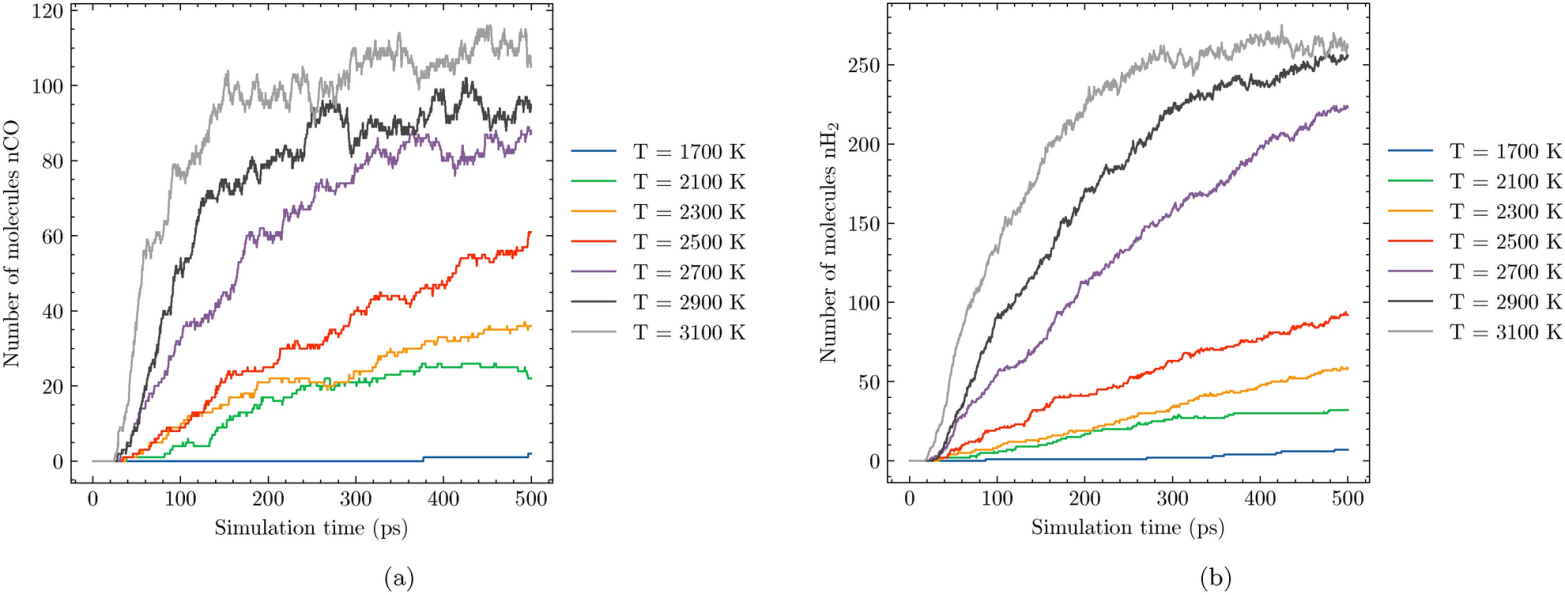
Evolution of CO (**a**) and $\hbox {H}_{2}$(**b**) molecules during the SCWG process in System 4 at different temperatures.

Water plays a crucial role in the SCWG process. To explore its influence, we compared system 4, which contains 82% water, with system 1, comprising 60% water. Figure 7 illustrates the formation of CO and molecular $\hbox {H}_{2}$ across varying temperatures. The results demonstrate that at lower temperatures, both systems exhibit relatively low levels of gaseous products, with their concentrations increasing as temperature rises. Notably, $\hbox {H}_{2}$ consistently outperforms CO in terms of molar fraction across all temperatures studied. At T = 3100 K, an interesting observation emerges: while the CO levels are comparable between system 1 and system 4, system 4 exhibits a significantly higher $\hbox {H}_{2}$ production rate compared to system 1. This suggests that increasing water content enhances hydrogen formation in SCWG reactions. This finding aligns with previous research on hydrothermal gasification of polyethylene by Ha et al.^37^, where elevated water content also promoted hydrogen production. The increased availability of water likely facilitates the breaking of carbon-hydrogen bonds, thereby enhancing the yield of molecular hydrogen. This underscores the importance of optimizing water content in SCWG processes to maximize hydrogen output and improve overall efficiency.

**Fig. 8:**
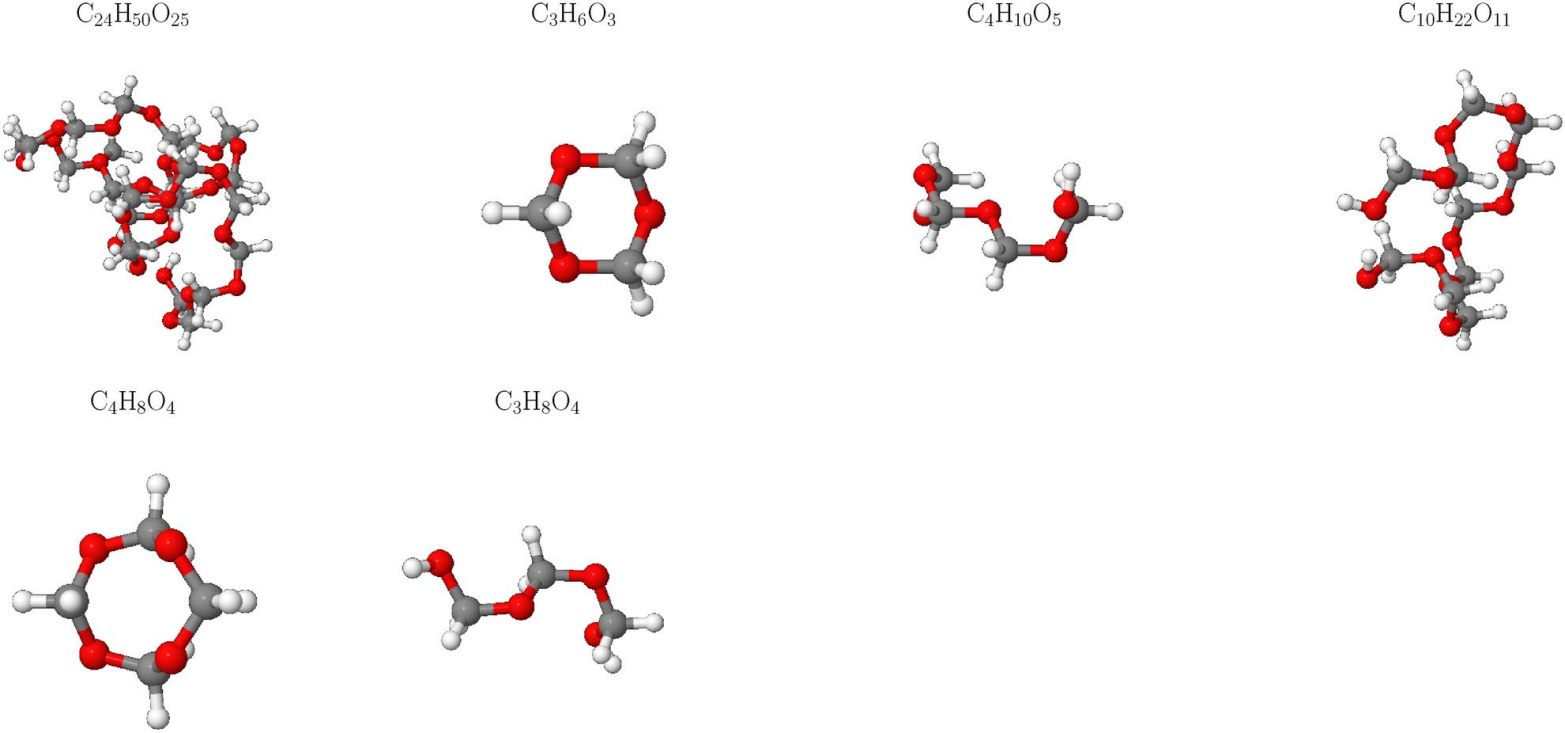
Snapshot of the largest product molecules with their chemical formula during the SCWG process of system 4 at temperature at 1400 K. The gray, white and red color represent for C, H and O atoms, respectively.

To further investigate the effects of varying water content on product formation during SCWG, we examined the largest product molecules in system 4 at a low temperature of T = 1400 K (Fig. 8). Our analysis revealed a striking similarity between system 4 and system 1, with both systems producing large organic fragments such as $\hbox {C}_{24}\hbox {H}_{50}\hbox {O}_{25}$ and $\hbox {C}_{10}\hbox {H}_{22}\hbox {O}_{11}$, along with smaller cyclic compounds like $\hbox {C}_{3}\hbox {H}_{6}\hbox {O}_{3}$. This observation suggests that, at lower temperatures, the influence of water content on fragment size is minimal, resulting in comparable decomposition patterns across systems. A more comprehensive information of the molecular fragment compositions in both system 1 and system 4 can be found in the Supplementary Information (Tables S2 and S3). Although there are slight differences in intermediate components between the two systems, they share large molecular modules due to the fact that the supercritical water gasification process is not yet complete in both cases.

At higher temperatures, however, the reaction becomes more complete, leading to the absence of fragments larger than C2. This indicates that temperature plays a dominant role in determining the extent of polymer degradation, with sufficient thermal energy enabling the breakdown of even the largest molecular structures into simpler components. The similarity in fragment formation at low temperatures across systems with differing water contents suggests that other factors, such as reaction kinetics and bond-breaking mechanisms, may exert greater control over product distribution under these conditions. As temperature increases, the enhanced availability of thermal energy likely facilitates more complete decomposition, reducing reliance on specific water content levels for achieving desired product distributions.

**Fig. 9:**
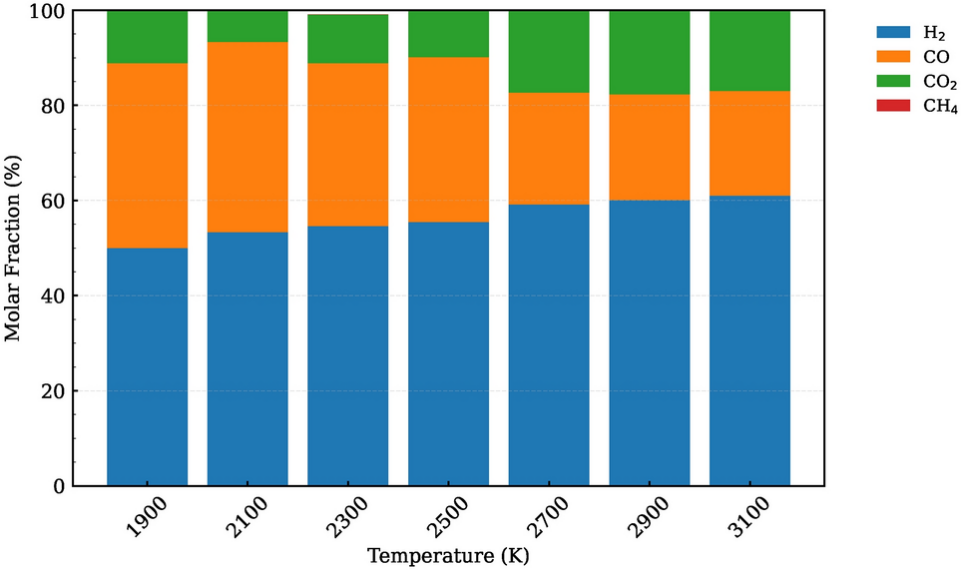
Gas fraction composition of the SCWG process for system 4 at different temperatures.

The gas molar fractions of major gaseous products generated during the SCWG process for system 4, characterized by an 82% water content, are illustrated in Fig. 9. The composition closely resembles that of system 1, with $\hbox {H}_{2}$ dominating the molar fraction, followed by CO and $\hbox {CO}_{2}$.

Interestingly, system 4 exhibits no methane ($\hbox {CH}_{4}$), whereas system 1 shows a small amount. This contrast highlights the influence of operational parameters on product distribution. Specifically, the absence of methane in system 4, which operates under higher water content conditions, aligns with experimental findings reported by Lu et al.^29^, who observed no methane formation at 95% water content. This suggests that elevated water content effectively inhibits methane formation while promoting hydrogen production. The enhanced oxidative environment facilitated by higher water content likely favors complete oxidation processes, thereby reducing the likelihood of methane formation.

It is worth mentioning that in Fig. 9, the molar fraction of hydrogen increases with rising temperature. Nevertheless, beyond 2700 K, the hydrogen fraction reaches its peak and ceases to grow further, suggesting that maximum hydrogen production occurs at a specific temperature threshold. Excessively high temperatures may not necessarily result in increased hydrogen gas yields, as observed in the data. Beyond 2700 K, the gasification reaction of POM plastics reaches near-complete conversion, indicating that further increasing the temperature primarily affects the reaction kinetics rather than enhancing the overall conversion. This highlights the importance of optimizing reaction conditions to achieve maximum hydrogen production.

In order to evaluate the impact of water content on gasification product distribution, a comparative analysis between system 1 (lower water content) and system 4 (higher water content) is conducted. Notably, system 4 exhibits a higher molar fraction of hydrogen compared to system 1. This observation can be attributed to the increased presence of water in system 4, which generates more hydroxyl and hydrogen radicals that promote hydrogen formation. Additionally, the absence of methane in system 4 signifies that the methanation reaction is outcompeted by gasification and water-gas shift reactions under higher water content conditions. Furthermore, our study reveals that system 4 exhibits a higher molar fraction of $\hbox {CO}_{2}$ compared to system 1. This observation can be explained by the increased prevalence of water in system 4, as it leads to a more significant proportion of water gas shift reaction products, resulting in the formation of greater amounts of $\hbox {CO}_{2}$. The relationship between water content and $\hbox {CO}_{2}$ production underscores the importance of optimizing hydration levels for efficient conversion during supercritical water gasification processes.

### Kinetic and activation energy

Understanding the reaction kinetics of the SCWG process is pivotal for optimizing process efficiency and achieving high yields of desired products. Reaction kinetics govern how rapidly reactants are converted into products, directly influencing both the economic viability and environmental footprint of the SCWG process. A widely used approach in similar research has been to analyze the carbon conversion rate^38,46^, which provides valuable insights into the kinetic behavior of the system.

The carbon conversion (CC) is quantified as a percentage, reflecting the efficiency of carbon transformation during gasification process. It is calculated by dividing the total number of carbon atoms converted into gaseous products by the initial number of carbon atoms in POM, denoted as $n_0$. The equation for carbon conversion is expressed as:

6 $$\begin{aligned} \text {Carbon Conversion} (\%) = \frac{n_{\textrm{CO}} + n_{\textrm{CO2}} + m \times n_{\textrm{C}_m\textrm{H}_n}}{n_0} \times 100\% \end{aligned}$$

In this formulation, $n_{\textrm{CO}}$, $n_{\textrm{CO}_{2}}$, and $n_{\textrm{C}_{m}\hbox {H}_n}$ represent the number of molecules of carbon monoxide ($\hbox {CO}$), carbon dioxide ($\hbox {CO}_{2}$), and other hydrocarbon gases, respectively. The variable $m$ signifies the number of carbon atoms in each hydrocarbon molecule, accounting for their contribution to the total carbon conversion.

One of the critical parameters derived from carbon conversion rate data is the activation energy ($E_a$) of the process. This study investigates $E_a$ values obtained through ReaxFF simulations, which provide insights into the energetic requirements for reaction initiation. By continuously monitoring the progression of carbon conversion over time, we were able to ascertain the reaction rate and its sensitivity to varying conditions. To analyze this dependency, a first-order reaction model was employed, as illustrated by Equation 7:

7 $$\begin{aligned} \frac{\text {d}[A]}{\text {d}t} = -k[A] \end{aligned}$$

In this equation, [A] represents the carbon conversion rate in the system at any given time t, and k is the rate constant that governs the reaction’s kinetics.

By fitting the data to the integral form of Eq. (7), we were able to determine the value of k for each temperature condition, thereby enabling us to assess the effects of temperature on the reaction rate. The obtained results allowed us to draw valuable insights into the mechanisms underlying the hydrothermal gasification process and its dependence on temperature.

The rate constant k in Eq. (7) obeys the Arrhenius relationship presented in Eq. (8).

8 $$\begin{aligned} k = Ae^{-\frac{Ea}{RT}} \end{aligned}$$

In Eq. (8), Ea is the activation energy, R is the gas constant and T is the absolute temperature.

**Fig. 10:**
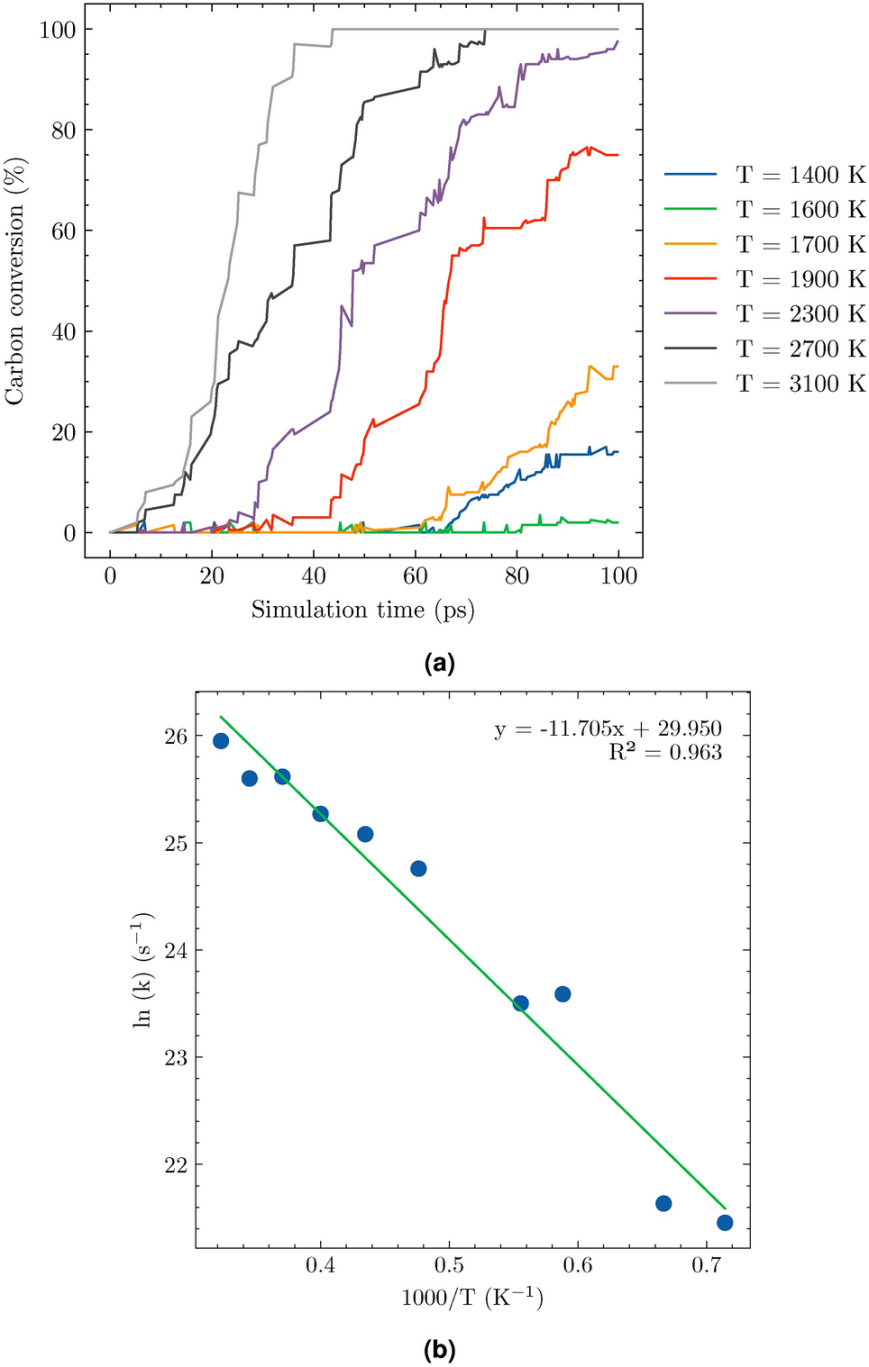
Carbon conversion of system 1 during SCWG process at different temperatures (**a**) and the plot of logrithm of rate constant as a function of the inverse temperatures (**b**).

Figure 10.a illustrates the variation in carbon conversion rates for system 1 across different temperatures. This parameter is crucial as it reflects the efficiency of carbon transformation during gasification. At a lower temperature of 1400 K, the carbon conversion rate is notably low, approximately 1%, indicating minimal reaction activity. As the temperature increases, there is a corresponding rise in the carbon conversion rate, demonstrating the significant influence of thermal energy on reaction kinetics. Interestingly, alongside this increase in conversion rates, the time required to achieve maximum conversion diminishes with higher temperatures. This inverse relationship underscores the importance of optimizing temperature settings to balance between achieving high conversion rates and minimizing process duration. These findings are consistent with prior studies on polyethylene gasification^37^, suggesting that similar thermodynamic principles govern the behavior of different polymer systems under supercritical water conditions.

Figure 10.b presents a plot of the natural logarithm of the rate constant ($\ln (k)$) versus the reciprocal of temperature ($1/T$) for system 1. This graph demonstrates a strong linear correlation, which is characteristic of the Arrhenius relationship. The slope of this linear plot, calculated as $-E_a/R$, allows for the determination of the activation energy ($E_a$) required for the reaction to occur. For system 1, the derived activation energy is 106 kJ/mol.

**Table 5:** Activation energy $E_a$ of the SCWG process of microplastics POM obtained from ReaxFF MD. Experimental value^29^ is added for comparasion.

System	Feedstock wt%	$E_a$ (kJ/mol)
1	40%	106 ± 8
2	27%	112 ± 10
3	23%	115 ± 7
4	15%	135 ± 11
Exp.^29^	5%	160

Table 5 presents activation energy ($E_a$) values for the SCWG of POM microplastics across four systems with varying feedstock percentages, along with an experimental value for comparison. As observed, $E_a$ increases as the feedstock weight percentage decreases, suggesting that higher water content lowers the activation energy required for the reaction. This trend indicates enhanced reaction efficiency at higher water levels, potentially due to improved solubility and catalytic effects facilitated by water.

System 1, with a 40% feedstock concentration (60% water), exhibits the lowest $E_a$ of 106 kJ/mol, while Systems 2 and 3 show slightly higher values at 112 and 115 kJ/mol for 27% and 23% feedstock, respectively. System 4, operating at 15% feedstock (85% water), demonstrates a significant rise to 135 kJ/mol. The experimental value of $E_a = 160$ kJ/mol at an extreme low feedstock concentration of 5% (95% water) further emphasizes this trend, highlighting the substantial increase in activation energy requirements under highly aqueous conditions.

The observed increase in activation energy with decreasing feedstock percentage aligns with findings from polyethylene gasification studies^37^. This relationship arises because higher water content can impede the diffusion of reactants and intermediates within the reaction medium, leading to slower overall reactions and thereby increasing the effective activation energy required for the process. In SCWG, water serves both as a solvent and a reactant. While it facilitates certain chemical transformations, excessive water content can create a more viscous or less mobile reaction environment, which hinders the movement of essential species and slows down reaction kinetics. This phenomenon is consistent with observations in polyethylene gasification^37^, where similar trends were noted under varying moisture conditions.

The activation energy trend observed in our MD simulations demonstrates excellent agreement with experimental data. Specifically, system 4, operating at 85% water content, exhibits an activation energy ($E_a$) of 135 kJ/mol, which closely aligns with the experimental value of 160 kJ/mol reported for a SCWG of POM at 95% water content^29^. This consistency underscores the validity and reliability of our simulation model in accurately predicting reaction energetics under SCWG conditions. The observed increase in $E_a$ from 135 kJ/mol to 160 kJ/mol as water content rises from 85% to 95% suggests a proportional relationship between hydration levels and activation energy requirements.

In order to reduce computational costs associated with simulating a system composed of nearly 18,000 atoms (at 95% water content), we did not directly perform simulations at this specific condition. Nonetheless, based on our findings for the 85% water content case, we anticipate that the activation energy in the 95% water content scenario would be slightly higher to the values observed in the 85% water content system. This extrapolation provides a reasonable estimate of the activation energy behavior at higher water contents.

POM exhibits a relatively low activation energy compared to other plastics, making it an attractive candidate for co-gasification processes. This characteristic facilitates easier reaction initiation and integration of POM with other materials, offering promising avenues for enhancing overall process efficiency. Co-gasification involves combining different waste streams, such as various types of plastics or biomass, to optimize energy recovery and reduce environmental impact. The low activation energy of POM enables it to serve as a reactive component in these mixtures, potentially accelerating reaction rates and improving yields of valuable gaseous products like $\hbox {H}_{2}$, $\hbox {CO}$, and $\hbox {CO}_{2}$. Moreover, incorporating POM into co-gasification systems could offer economic benefits by utilizing otherwise hard-to-recycle plastic waste as a supplementary feedstock. This approach not only addresses the growing challenge of plastic pollution but also contributes to sustainable energy production.

Compared to other waste management methods such as hydrothermal carbonization (HTC) and liquefaction (HTL), SCWG generally requires more energy due to the gasification reactions occurring at higher temperatures. However, this method offers a significant advantage by enabling the conversion of polymeric waste materials (POM plastics) into hydrogen gas. Hydrogen is a valuable resource in various industrial applications, including clean energy production through fuel cells and chemical feedstocks. By optimizing SCWG reaction conditions and advancing technologies for better hydrogen recovery, we can enhance the efficiency of this process while reducing energy consumption and contributing to both waste management and sustainable energy generation.

## Conclusions

This study presents an in-depth analysis of supercritical water gasification (SCWG) for Polyoxymethylene (POM), addressing the critical issue of microplastics pollution. Through ReaxFF molecular dynamics simulations, we investigate key factors influencing SCWG, including temperature, water content, carbon conversion efficiency, and product yields. Our findings validate the effectiveness of ReaxFF MD in modeling these reactions and confirm that hydrogen, carbon monoxide, and carbon dioxide are the primary products of POM gasification.

Temperature significantly impacts the efficiency of the process, with higher temperatures enhancing carbon conversion rates. Additionally, water content influences hydrogen production while slightly increasing the activation energy barrier, underscoring the importance of precise process control. Our computational approach yielded activation energies ranging between 106 and 135 kJ/mol for the supercritical water gasification of POM plastics. This calculated range demonstrates excellent agreement with experimental findings, which report an activation energy of 160 kJ/mol^29^. The strong correspondence between simulation results and experimental data highlights the reliability and predictive capacity of our computational approach for guiding process design and optimization in sustainable plastic waste management strategies.

It is interesting to that the gasification of POM has relatively lower activation energy compared to other plastics, such as polyethylene, makes it highly suitable for co-gasification processes. This characteristic opens avenues for efficient waste management and energy recovery when combined with other plastic types. Future research directions include investigating interactions between POM and other plastics, optimal blending ratios for energy recovery, and the impact of varying operational parameters. Additionally, exploring catalyst utilization to enhance reaction efficiency in co-gasification systems presents a promising area for further study.

In conclusion, this research highlights the importance of integrating computational modeling with empirical studies to deepen our understanding of SCWG processes. The molecular insights gained offer a foundation for designing optimized systems that convert plastic waste into valuable energy resources, thereby supporting environmental conservation and renewable energy initiatives.

## Supplementary Information

Supplementary Information.

## Data Availability

The datasets generated and/or analyzed during the current study are available from the corresponding author on reasonable request.
