# Supplementary material for: Molecular insights into supercritical water gasification process of polyoxymethylene plastics
Source: Sci Rep. 2025 Mar 18;15:9382. doi: 10.1038/s41598-025-93887-5 (PMC11920286; doi:10.1038/s41598-025-93887-5)
Supplement: Supplementary file 1 — Supplementary Information. [file 41598_2025_93887_MOESM1_ESM.pdf]

Supporting Information: Molecular insights into supercritical water gasification process of polyoxymethylene plastics

Do Tuong Ha,*,† Dao Trinh,‡ Thi Be Ta Truong,† and Thuat T. Trinh*,¶

†*Faculty of Applied Sciences, Ton Duc Thang University, Ho Chi Minh city, Vietnam*

‡*Laboratoire des Sciences de l'Ingénieur pour l'Environnement, LaSIE UMR 7356 CNRS, La Rochelle Université, Avenue Michel Crépeau, 17000 La Rochelle, France*

¶*Porelab, Department of Chemistry, Norwegian University of Science and Technology, Høgskoleringen 5, 7491-Trondheim, Norway*

E-mail: dotuongha@tdtu.edu.vn; thuat.trinh@ntnu.no

In the Supplementary Information, we present the parameters for the ReaxFF simulations, independent run tests, molecular fragments, and the evolution of the largest molecules throughout the reaction.

Table S1: Parameters for the ReaxFF molecular dynamics simulations.

**Parameter**	**Value/Range**
Potential function	Developed by Vashisth et al.[S1]
Validation methods	Experimental data, DFT calculations,[S1] other systems[S1–S4]
Simulation software	ReaxFF with LAMMPS[S5]
Equilibration time	5 ps at 300 K in NVT ensemble[S2,S3]
Production run temperatures	1400 K to 3100 K[S3,S4,S6]
Heating rate	20 K/ps[S2,S3]
Time step	0.25 fs[S2,S3]

Table S2: Top 10 fragments of system 1 at T = 1400 K.

Formula	Mass (g/mol)	Average Count
H_2O	18.0	498.0
CH_2O	30.0	92.0
$C_3H_6O_3$	90.0	18.0
$C_4H_8O_4$	120.0	2.0
CH_4O	32.0	2.0
$C_{22}H_{44}O_{22}$	660.2	1.0
$C_7H_{16}O_7$	212.1	1.0
$C_7H_{15}O_7$	211.1	1.0
$C_2H_6O_3$	78.0	1.0
$C_2H_4O_2$	60.0	1.0

Table S3: Top 10 fragments of system 4 at T = 1400 K.

Formula	Mass (g/mol)	Average Count
H_2O	18.0	1495.0
CH_2O	30.0	110.0
$C_3H_6O_3$	90.0	11.0
CH_2O_2	46.0	2.0
C_2H_5O	45.0	2.0
$C_{24}H_{50}O_{25}$	738.3	1.0
$C_{10}H_{22}O_{11}$	318.1	1.0
$C_4H_{10}O_5$	138.1	1.0
$C_4H_8O_4$	120.0	1.0
$C_3H_8O_4$	108.0	1.0

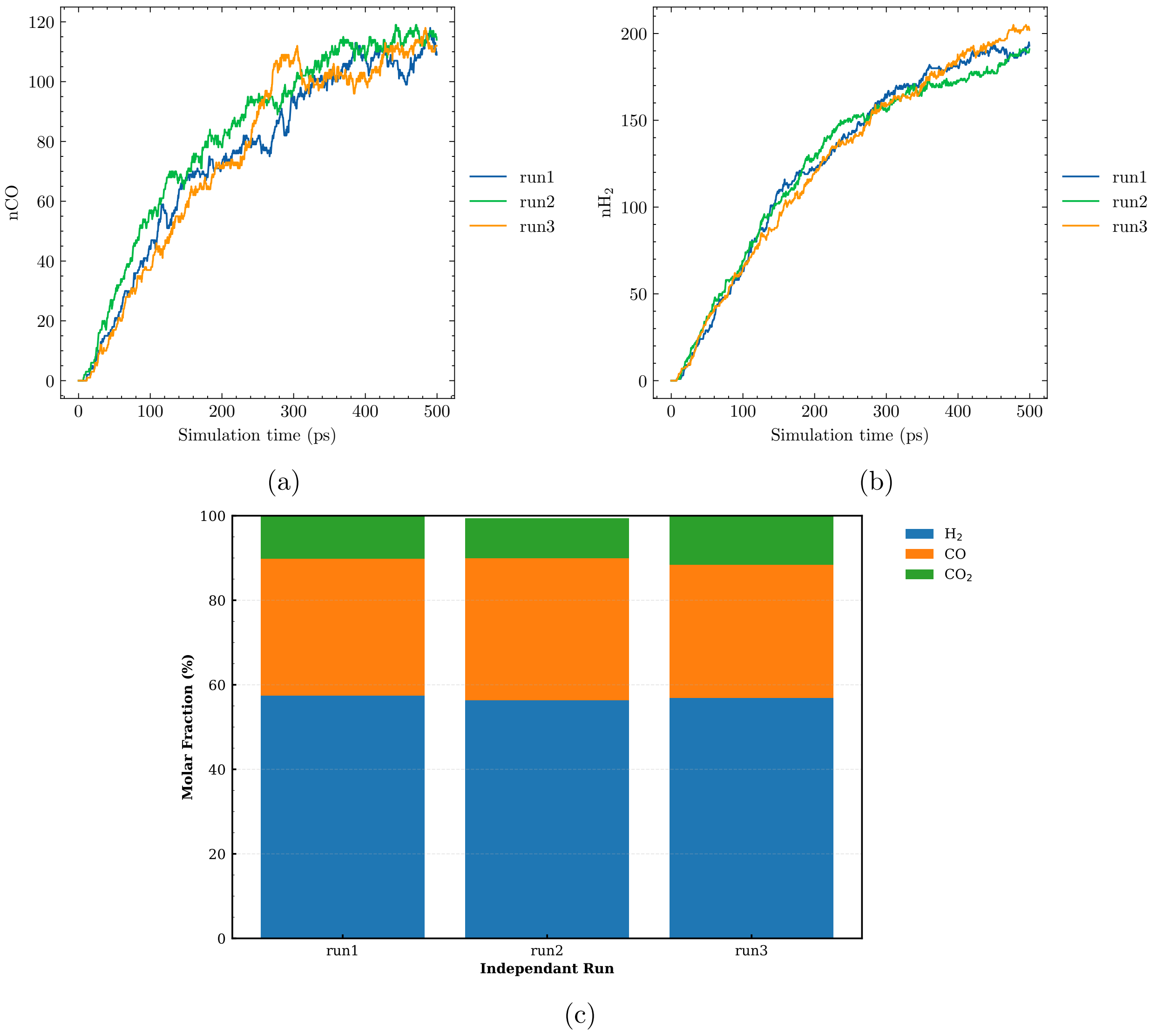

Figure S1: Evolution of the number of CO (a) and H_2 (b) molecules during three independent runs. The molar gas fraction (c) exhibits nearly identical profiles across all runs, indicating reproducibility of the reaction dynamics.

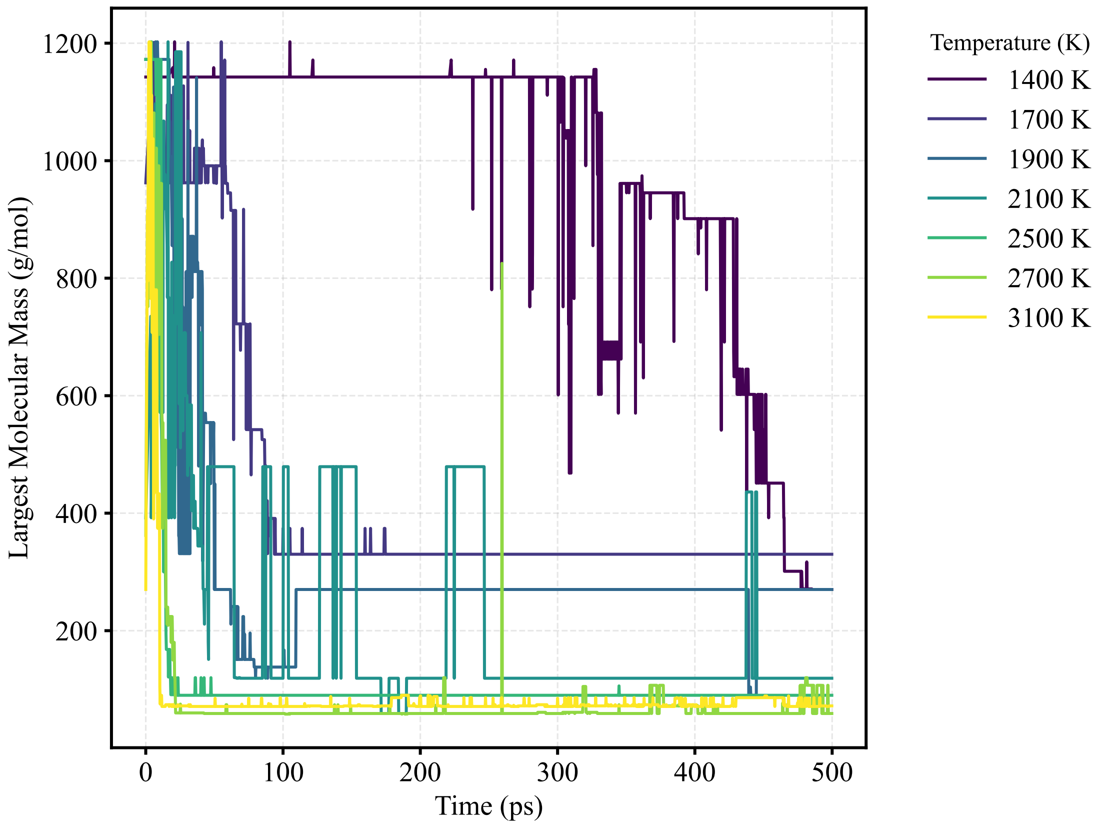

Figure S2: Time evolution of the largest molecular mass of a molecular fragment in System 1 during ReaxFF simulations at different temperatures. The plot illustrates the decomposition processes within the system.

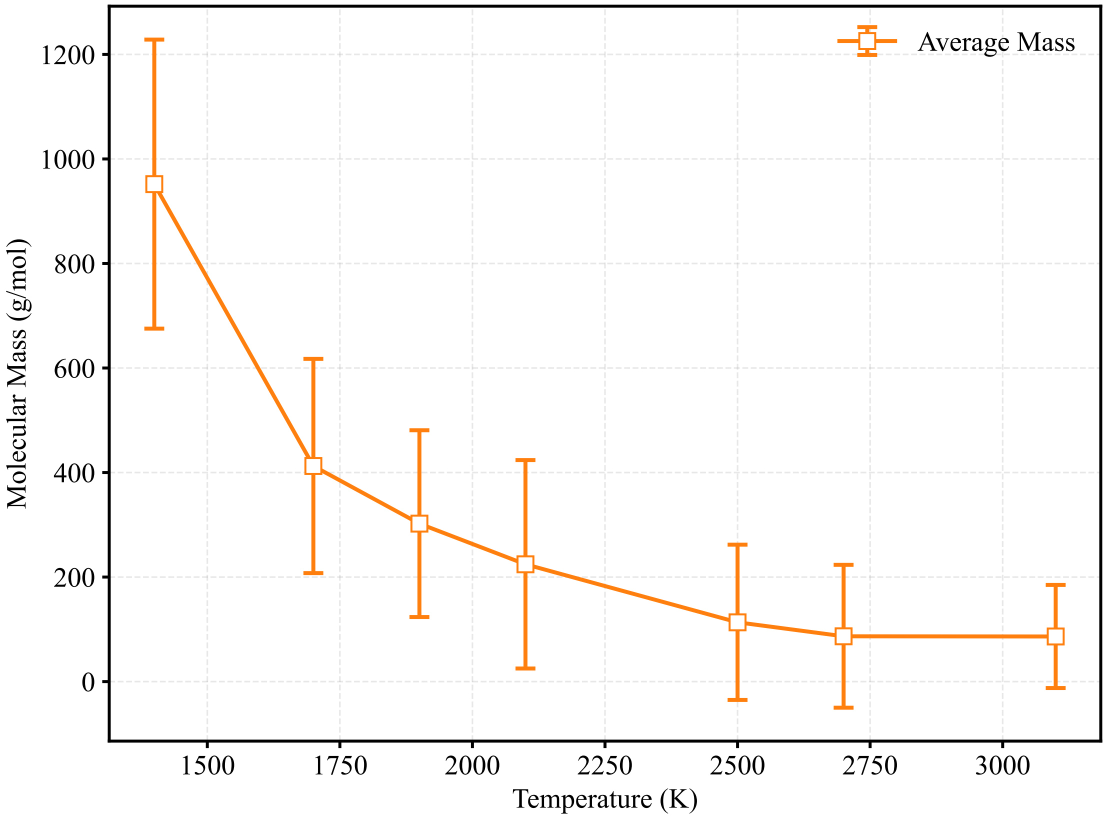

Figure S3: Variation of the average molecular mass of System 1 with reaction temperature. Higher temperatures lead to a lower average molecular mass, indicating increased fragmentation and decomposition.

References

(S1) Vashisth, A.; Ashraf, C.; Zhang, W.; Bakis, C. E.; van Duin, A. C. Accelerated ReaxFF Simulations for Describing the Reactive Cross-Linking of Polymers. *J. Phys. Chem. A* **2018**, *122*, 6633–6642.

(S2) Ha, D. T.; Tran, K.-Q.; Trinh, T. T. New insights into the hydrothermal carbonization process of sewage sludge: A reactive molecular dynamics study. *Fuel* **2024**, *361*, 130692.

(S3) Ha, D. T.; Tong, H. D.; Trinh, T. T. Insights into hydro thermal gasification process of microplastic polyethylene via reactive molecular dynamics simulations. *Scientific Reports* **2024**, *14*, 18771.

(S4) Truong, T. B. T.; Ha, D. T.; Tong, H. D.; Trinh, T. T. ReaxFF molecular dynamics studies on the impact of reaction conditions in polystyrene conversion through hydrothermal gasification. *Chemical Engineering Journal Advances* **2025**, *22*, 100716.

(S5) Thompson, A. P.; Aktulga, H. M.; Berger, R.; Bolintineanu, D. S.; Brown, W. M.; Crozier, P. S.; in 't Veld, P. J.; Kohlmeyer, A.; Moore, S. G.; Nguyen, T. D.; Shan, R.; Stevens, M. J.; Tranchida, J.; Trott, C.; Plimpton, S. J. LAMMPS - a flexible simulation tool for particle-based materials modeling at the atomic, meso, and continuum scales. *Comput. Phys. Commun.* **2022**, *271*, 108171.

(S6) Liu, X.; Wang, T.; Chu, J.; He, M.; Li, Q.; Zhang, Y. Understanding lignin gasification in supercritical water using reactive molecular dynamics simulations. *Renew. Energ.* **2020**, *161*, 858–866.
